# Sex-Specific Patterns of Taste Dysfunction, Their Relationships with α-Synuclein Profiling, and Supervised Learning-Based Diagnosis in Parkinson’s Disease (PD)

**DOI:** 10.3390/ijms27094048

**Published:** 2026-04-30

**Authors:** Melania Melis, Fabrizio Angius, Lala Chaimae Naciri, Giorgia Sollai, Silvia Deligia, Giuseppe Fenu, Paolo Mellino, Beatrice Pinna, Roberto Crnjar, Anna R. Carta, Giovanni Cossu, Iole Tomassini Barbarossa

**Affiliations:** 1 Department of Biomedical Sciences, University of Cagliari, 09042 Monserrato, Italy; fangius@unica.it (F.A.); l.naciri@studenti.unica.it (L.C.N.); gsollai@unica.it (G.S.); silvia.deligia@unica.it (S.D.); crnjar@unica.it (R.C.); acarta@unica.it (A.R.C.); tomassin@unica.it (I.T.B.); 2 Department of Neurology, AO Brotzu, 09134 Cagliari, Italy; giusefenu@gmail.com (G.F.); pmellino95@gmail.com (P.M.); beatricepinna@hotmail.com (B.P.); giovannicossu1@gmail.com (G.C.)

**Keywords:** taste perception, supervised learning, salivary oligomeric α-syn, α-synuclein genetic variants

## Abstract

Taste impairment is a little-known non-motor Parkinson’s disease (PD) feature with potential diagnostic value. However, its biological basis and sex-specific patterns remain unclear. We combined psychophysical taste testing, salivary α synuclein (αsyn) profiling, genotyping of four *SNCA* polymorphisms, and Supervised Learning (SL) within a unified, sex-aware analytical framework to analyze sensory, molecular, and genetic correlates of gustatory dysfunction in 99 PD patients and 60 healthy controls. Overall taste identification was markedly reduced in PD, independently of sex. However, males and females showed distinct taste quality alterations: females preserved sour recognition, while males showed marked citric acid misidentification. SL modeling achieved high accuracy, revealing that the inability to perceive saltiness was most informative overall, astringency misidentification strongly predicted female PD, and sour misidentification characterized male PD. Salivary oligomeric αsyn showed a significant sex × diagnosis interaction, being elevated only in PD females, specifically those failing to identify astringency. Genotype–phenotype analyses revealed sex-dependent associations between *SNCA* variants (*rs356219*, *rs181489*, and *rs2583988*) and astringency recognition. These findings demonstrated that sex critically shapes the interplay between taste dysfunction, peripheral αsyn biology, and *SNCA* genetics in PD, supporting sex-aware chemosensory phenotyping and the development of precision taste-based biomarkers.

## 1. Introduction

Parkinson’s disease (PD) is a progressive neurodegenerative disorder characterized not only by its hallmark motor symptoms—bradykinesia, rigidity, resting tremor, and postural instability—but also by a broad spectrum of non-motor manifestations that substantially impact quality of life [1,2,3,4]. These include cognitive impairment, sleep disturbances, autonomic dysfunction, psychiatric symptoms, and hyposmia, reflecting the complex and heterogeneous nature of PD pathophysiology [4,5,6,7]. Converging evidence indicates that many factors have been associated with the risk of PD. Among them, variations in the concentrations of α-synuclein forms, i.e., total (tot-αsyn), oligomeric (o-αsyn), and phosphorylated (p-αsyn), in peripheral fluids, including blood, saliva, and cerebrospinal fluid, have been identified as PD biomarkers, potentially indicative of the neuropathological outcome [8,9]. Specifically, o-αsyn has high pathological relevance in PD, being recognized as one of the most toxic post-translational species of the protein [10]. Salivary o-αsyn levels yielded strong discriminative value in PD [11], showing higher concentration in patients compared to healthy controls (HC). This evidence supports the involvement of salivary levels of o-αsyn in PD, while data on total quantities are inconsistent [12]. Furthermore, salivary α-synuclein levels are influenced by specific α-synuclein (SNCA) single-nucleotide polymorphisms (SNPs) [13,14,15]. Several *SNCA* SNPs, including *rs356219*, *rs181489* TT, *rs2583988* CC, *rs356186*, and *rs356165*, have been repeatedly associated with an elevated PD risk across populations [13,14,16,17,18]. Moreover, variants within SNCA have been linked not only to PD onset, but also to differences in clinical presentation and progression, although their contributions to symptom-level heterogeneity remain modest and inconsistently replicated [14]. In addition, rare pathogenic SNCA mutations and gene multiplications (such as duplications or triplications) cause autosomal-dominant forms of PD, as do common SNPs that increase disease risk and may contribute to clinical heterogeneity [19,20,21,22].

Growing interest has emerged around chemosensory impairment, particularly olfactory and taste dysfunction, as early indicators of PD [23,24,25,26,27]. Taste deficits, although less studied than olfactory loss, may reflect early involvement of peripheral sensory pathways or central gustatory processing networks [28]. However, previous works have shown inconsistent findings, and few studies have examined how genetic factors interact with taste impairment profiles in PD [1,29]. Our recent multi-dimensional study filled this gap by demonstrating that taste deficits in PD are modality-specific, with saltiness and astringency being the most affected, and that specific *SNCA* genotypes modulate these sensory abnormalities [28]. These findings supported the hypothesis that taste anomalies in PD are both functional and genetically determined, possibly providing a prodromal or early biomarker of the disease.

An important yet underexplored dimension in this context is biological sex. A growing body of literature indicates that PD differs substantially between males and females in terms of epidemiology, risk factors, motor and non-motor manifestation patterns, rate of progression, and treatment responses. A recent narrative review confirmed that females exhibit a higher risk of disabling motor complications and non-motor fluctuations, while males more frequently develop cognitive impairment, gait disturbances, and postural instability [30]. Large-scale cohort analyses also showed that sex influences the burden of non-motor symptoms, with females reporting more anxiety and autonomic dysfunction, while males exhibit greater cognitive decline [31]. Furthermore, longitudinal data have suggested that disease progression trajectories differ by sex, with females showing slower deterioration in cognitive, sleep, and postural-gait metrics [32].

Given the sexual dimorphism observed in dopaminergic neurobiology, hormonal influences (e.g., estrogenic neuroprotection), autonomic physiology, salivary function, and immune responses—all of which may affect gustatory processing—investigating sex differences in taste biomarkers is both biologically plausible and clinically relevant. To date, no study has systematically evaluated how sex, taste impairments, *SNCA* polymorphisms, salivary levels of α-synucleins, and PD diagnosis intersect within a unified analytical framework. Extending research in this direction could therefore fill a significant gap in the field. Furthermore, understanding sex-specific patterns of sensory dysfunction and their functional and genetic underpinnings could refine early diagnostic models, improve disease-stratification strategies, and ultimately contribute to personalized approaches to PD management.

The primary aim of this work was to analyze the relationships between taste function, salivary levels of α-synuclein species, α-synuclein gene (*SNCA*) variants, and sex in PD patients and HCs. Sex-related differences in the diagnostic significance of taste stimuli were analyzed using Supervised Learning (SL). Taste perception in PD patients and HCs of both sexes was determined as the identification of taste stimuli: sweet, salty, sour, bitter, including astringency, a complex oral-sensory sensation that occurs when stimuli interact with salivary proteins [29,30,31]. The α-synuclein gene (*SNCA*) SNPs chosen in this work were those previously associated with disease susceptibility [8,9,10,11,12,24,32] and taste deficits in PD patients [24].

## 2. Results

### 2.1. Sex-Related Differences in Taste Perception and Its Diagnostic Significance

**Figure 1:**
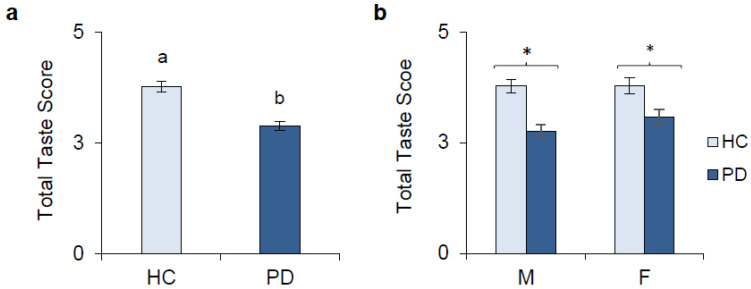
Total taste score determined in HCs and PD patients (**a**). The same data broken down by sex (**b**). HCs (*n* = 60): male (*n* = 32) and female (*n* = 28); PD patients (*n* = 99): male (*n* = 60); female (*n* = 39). Data are reported as mean values ± SE. Different letters indicate significant difference (H_(1,N=159)_ = 23.537; *p* < 0.0001; Kruskal–Wallis test). * denotes a significant difference (*p* < 0.014; Holm test subsequent Factorial General Linear Model HC3).

The mean value ± SEM of the total taste score (number of total identified strips) in PD patients was significantly lower than that in HCs (H_(1,N=159)_ = 23.537; *p* < 0.0001; Kruskal–Wallis test) (Figure 1a). When data were analyzed after gender stratification, the mean value ± SEM of the total taste score of male and female PD patients was significantly lower than that of male and female HCs (*p* < 0.001 and *p* = 0.014; Holm test, subsequent Factorial General Linear Model HC3) (Figure 1b).

**Figure 2:**
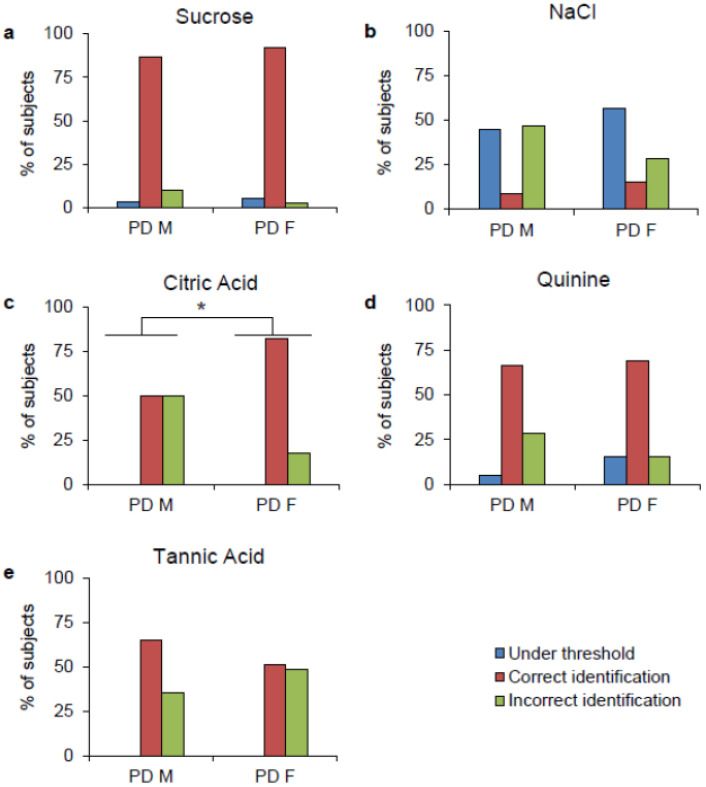
Distribution of male and female PD patients who perceived no taste (under threshold), correctly recognized the taste (correct identification), or stated a different quality (incorrect identification) for each taste stimulus: (**a**) sucrose, (**b**) NaCl, (**c**) citric acid, (**d**) quinine, and (**e**) tannic acid. * denotes a significant difference between male PD patients and female PD patients (χ^2^ = 10.374, *p* = 0.0013; Fisher’s exact test). Male PD patients (*n* = 60), female PD patients (*n* = 39).

Sex-related differences in the taste perception of PD patients are shown in Figure 2. For each taste stimulus (sucrose, NaCl, citric acid, quinine, and tannic acid), the distributions of both male and female PD patients who perceived no taste (indicated as under threshold), those who correctly recognized the taste (indicated as correct identification), or those who declared a different quality (indicated as incorrect identification) are shown. Fisher’s exact test showed similar patterns between the two groups when subjects tasted sucrose, NaCl, quinine, and tannic acid (*p* > 0.05) (Figure 2a,b,d,e). Differently, male PD patients and female PD patients showed different sensitivities when tasting the citric acid stimulus (χ^2^ = 10.374, *p* = 0.0013, Fisher’s exact test) (Figure 2c); 82% of females and 50% of males showed a correct identification. No difference was found between male and female HCs (*p* > 0.05).

**Figure 3:**
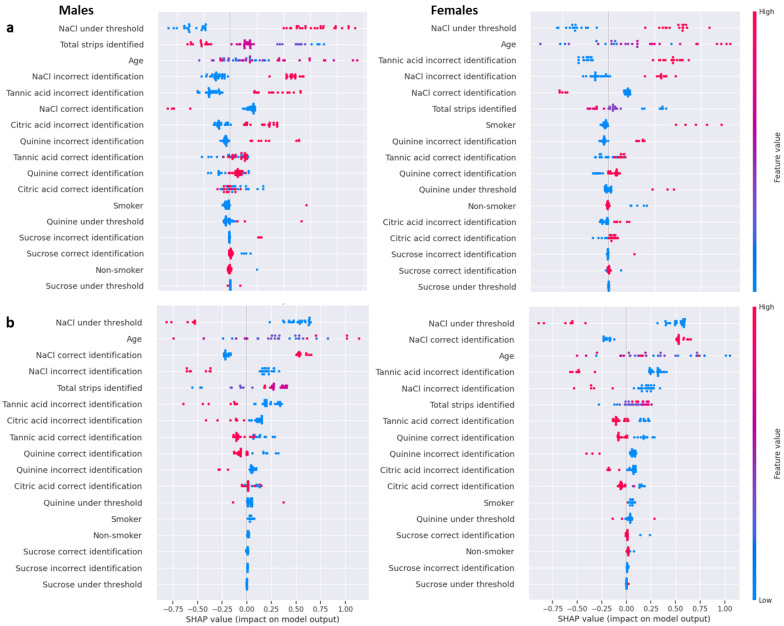
SHAP summary plots showing the importance and impact of features on the CatBoost classifier to predict PD patients (**a**) or HCs (**b**) differentiated by sex. The importance of the features (from top to bottom) is shown on the left of each Y-axis; the impact on the model output (SHAP value) is shown on the X-axis. Each point in the plot is a SHAP value of a feature, and its position on the X-axis shows the prediction score for the class. The feature value is indicated by the color of the line to the right of each graph: low value (blue), medium value (violet), and high value (red).

Sex-related differences in the diagnostic significance of taste stimuli were analyzed by using the SHAP algorithm, which allowed us to obtain an overview of the most important features and their impact in distinguishing between PD patients and HCs differentiated by sex. The high evaluation metrics (91% training accuracy, 91% training macro-averaged F1-score, 84% testing accuracy, and 82% testing macro-averaged F1-score) showed that the CatBoost algorithm achieved very good performance. Figure 3 shows the SHAP summary plots showing the importance and impact of features to predict PD patients (a) or HCs (b) differentiated by sex. Specifically, the NaCl under threshold was the first feature in order of importance for the four classes. High values strongly and positively correlated with PD prediction without differentiation by sex (Figure 3a), while low values pushed the model toward HC prediction (Figure 3b). The feature “NaCl correct identification” was positively correlated with HC classes and ranked second and third for female HCs and male HCs, respectively, while it was negatively correlated and ranked fifth and sixth for the female and male PD classes. It is important to note that the “total number of identified strips” feature was the second most negatively correlated with male PD predictions, while it was less important (sixth) to push the model toward female PD. This feature was also less important and positively correlated with both male and female HC prediction. Among the features characterizing the perception of a single taste stimulus, high values of “Tannic acid incorrect identification” were found to be strongly and positively correlated with female PD prediction, third in terms of importance, while they ranked fifth for the male PD class. It also negatively correlated with female and male HC classes, for which it ranked fourth and sixth, respectively. Notably, the “Citric acid incorrect identification” strongly and positively correlated with male PD prediction, as shown by SHAP plots of both male PD patients and male HCs. “Citric acid correct identification” also impacted the model for the male PD prediction, with low values (blue) that pushed the model toward this class. The “Quinine incorrect identification” feature showed an important impact on male and female PD predictions; high values positively correlated with this class. Interestingly, the “Smoker” feature had a high and positive impact to push the model toward the female PD class, while it had a low impact on predictions of other classes.

### 2.2. Sex-Related Differences in Salivary Levels of α-Synuclein Species and Their Effect on Taste Perception

**Figure 4:**
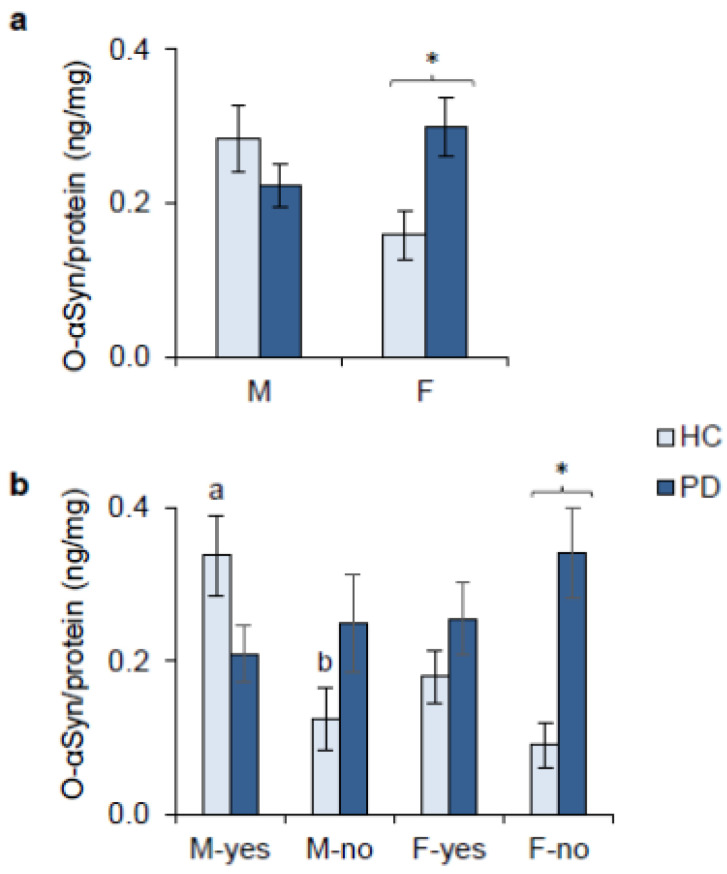
Salivary concentrations of o-αsyn determined in male and female patients with Parkinson’s disease (PD) and healthy controls (HCs) (**a**). Salivary concentrations of o-αsyn determined in male and female patients with Parkinson’s disease (PD) and male and female healthy controls (HCs) who correctly recognized tannic acid (M-yes and F-yes) and those of PD patients and HCs who did not recognize tannic acid (M-no and F-no) (**b**). Data are reported as mean values ± SE of concentrations normalized per mg of total salivary proteins. Male PD patients (*n* = 49), female PD patients (*n* = 37), male HCs (*n* = 31), and female HCs (*n* = 25). * or different letters indicate a significant difference (*p* < 0.042; Holm test subsequent Factorial General Linear Model HC3).

The BCA and ELISA methods allowed us to determine the salivary concentrations of total proteins, tot-αsyn, p-αsyn, and o-αsyn, respectively, in PD patients and HCs divided by sex. The Factorial General Linear Model HC3 revealed a significant two-way interaction of the sex × PD patients/HCs group only for the concentration values of o-αsyn (F _(1,138)_ = 7.855; *p* = 0.006) (Figure 4a). In females, pairwise comparison showed a significantly higher salivary concentration of o-αsyn in PD patients than in HCs (*p* = 0.022; Holm test subsequent Factorial General Linear Model HC3). No other differences were found (*p* > 0.05).

When the o-αsyn concentration data were further divided into subgroups based on taste discrimination, i.e., males and females who correctly recognized the tannic acid (M-yes and F-yes) and males and females who did not recognize tannic acid (M-no and F-no), post hoc comparison showed a significantly higher salivary concentration of o-αsyn in PD F-no than in HC F-no, and in HC M-yes than in HC M-no (*p* = 0.011 and *p* = 0.042; Holm test subsequent Factorial General Linear Model HC3) (Figure 4b).

No differences were found in the concentration of tot-αsyn, p-αsyn, or o-αsyn/tot-αsyn in PD patients and HCs related to sex or other taste stimuli (*p* > 0.05). 

**Figure 5:**
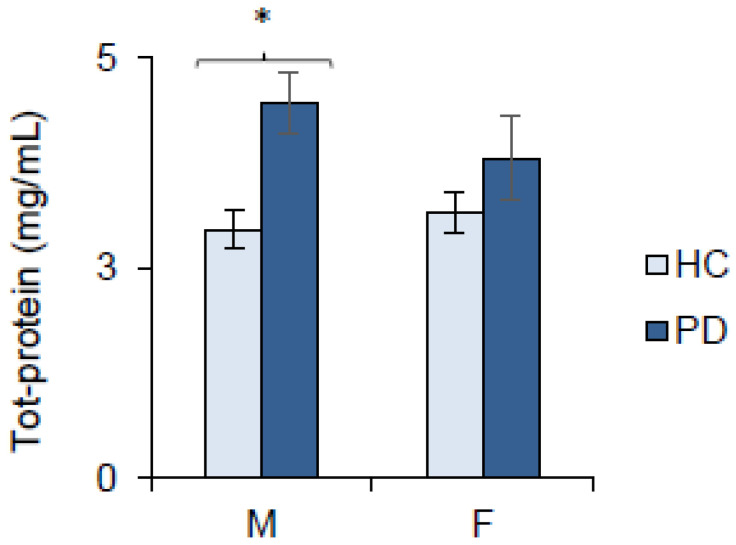
Salivary concentrations of the total proteins determined in male and female patients with Parkinson’s disease (PD) and healthy controls (HCs). Data are reported as mean values ± SE. Male PD patients (*n* = 49), female PD patients (*n* = 37), male HCs (*n* = 31), and female HCs (*n* = 25). * indicates a significant difference (*p* < 0.005; Holm test subsequent Factorial General Linear Model HC3).

The same analysis performed for salivary total proteins showed that the amount of total proteins in male PD patients was significantly higher than in male HCs (*p* < 0.005; Holm test subsequent Factorial General Linear Model HC3), while no differences were found between female PD patients and female HCs or females and males with PD (*p* > 0.05) (Figure 5).

### 2.3. Sex-Related Differences in the Effect of SNCA Polymorphisms (SNPs) on Taste Perception

Genotype distribution and allele frequencies of the *rs356219*, *rs181489*, *rs2583988*, and *rs356186* SNPs of SNCA in male and female PD patients and HCs are shown in Table S1. No differences were found between male and female PD patients and HCs according to the genotype distribution and allele frequency of *rs356219*, *rs181489*, *rs2583988*, and *rs356186* SNPs of *SNCA* (χ^2^ < 2.195; *p* > 0.334, Fisher’s method).

**Figure 6:**
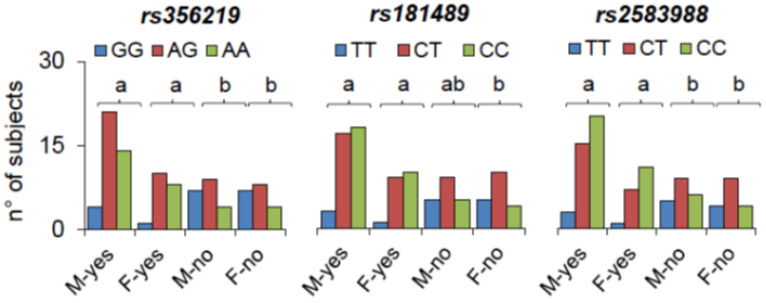
Genotype distributions of the *rs356219*, *rs181489*, and *rs2583988* SNPs of SNCA in male and female PD patients who correctly recognized the tannic acid stimulus (M-yes and F-yes) and those of male and female PD patients who did not recognize the tannic acid stimulus (M-no and M-no). Different letters indicate significant differences for population pair (χ^2^ > 5.969, *p* < 0.049; Fisher’s method).

The molecular analysis at the *rs356219*, *rs181489*, *rs2583988*, and *rs356186* SNPs of *SNCA* allowed us to determine the genotype distribution and allele frequency of male and female PD patients who correctly recognized the taste (M-yes and F-yes) and of those who did not (M-no and F-no). Figure 6 shows the data of participants who tasted the tannic acid stimulus. Fisher’s exact test showed that the four groups, M-yes, F-yes, M-no and F-no, differed statistically based on the genotype distributions of the *rs356219*, *rs181489*, and *rs2583988* SNPs of SNCA (*rs356219*: χ^2^ = 8.062, *p* = 0.018; *rs181489*: χ^2^ = 7.484, *p* = 0.024; *rs2583988*: χ^2^ = 7.339, *p* = 0.025; Fisher’s method) and allelic frequencies (*rs356219*: χ^2^ = 7.663, *p* = 0.021; *rs181489*: χ^2^ = 7.339, *p* = 0.025; *rs2583988*: χ^2^ = 7.798, *p* = 0.021; Fisher’s method). Pairwise comparisons discriminated M-yes from M-no and F-yes from F-no in the *rs356219* and *rs2583988* SNPs (χ^2^ > 5.969, *p* < 0.049, Fisher’s method). M-yes and F-yes had a high frequency of subjects with at least one allele A (90% and 95%, respectively) in the *rs356219* locus, and a high frequency of subjects with at least one allele C (92% and 94%, respectively) in the *rs2583988* locus, whereas M-no and F-no had a high frequency of subjects with at least one allele G (80% and 82%, respectively) in the *rs356219* locus, and a similar frequency of genotypes CC and TT (M-no: 25% and 30%; F-no: 23%) in the *rs2583988* locus. Pairwise comparisons also discriminated F-yes and F-no, in the *rs181489* SNP (χ^2^ = 6.667, *p* = 0.036, Fisher’s method). F-yes had a high frequency of subjects with at least one allele C (95%), whereas F-no had a high frequency of subjects with at least one allele T (79%). Interestingly, M-yes and M-no did not differ based on the genotype distribution, having a high frequency of subjects with at least one allele C (92% and 74%, respectively). No differences were found when participants tasted the other stimuli.

## 3. Discussion

In this study, integrated psychophysical taste testing, salivary α-synuclein (α-syn) profiling, SNCA genotyping, and SL were used in a single, sex-aware analytical framework to interrogate gustatory dysfunction in PD. Our results showed that gustatory dysfunction in PD is not only a robust non-motor feature but also can exhibit sex-specific patterns that could have clinical and biological significance. Three convergent messages emerge. First, PD was associated with a marked reduction in overall taste identification, as evidenced by lower total taste scores compared with healthy controls, an effect that persisted even after stratification by sex. This supports the hypothesis that gustatory dysfunction is a systemic feature of PD rather than a secondary consequence of demographic or behavioral factors. Second, a sex-specific pattern was observed within single-quality evaluations. Male and female PD patients showed a clear divergence for sour identification, with females showing relatively preserved recognition compared with males. Additionally, the inability to detect salty taste emerged as a shared vulnerability across sexes and appears to be a prominent feature of PD gustatory phenotype. Misidentification of tannic acid (astringency) contributed to PD classification in females, while misidentification of citric acid favored PD classification in males. Thus, alterations in astringency and sour perception further differentiated males and females, suggesting that PD can affect multiple chemosensory and somatosensory pathways in a sex-dependent manner. Overall, these findings indicate that gustatory dysfunction in PD is not uniform across taste modalities and that sex modulates both the expression and salience of these deficits. Third, salivary o-αsyn showed sex-dependent differences. In female PD patients, o-αsyn levels were higher relative to HCs, and specifically related to astringency misidentification. Conversely, no differences were found in males between PD patients and HCs, suggesting divergent peripheral biomarker profiles between sexes. These observations suggest a possible link between peripheral α-syn biology and altered oral sensory processing, which may be particularly relevant in females. Finally, genotype–phenotype analyses showed sex-dependent associations between *SNCA* variants (*rs356219*, *rs181489*, *rs2583988*) and the ability to recognize astringency. Taken together, these results extend current knowledge by demonstrating that taste phenotypes, salivary α-syn species, and common *SNCA* SNPs intersect in sex-specific, measurable, and diagnostically useful ways at the individual level.

Taste dysfunction is a recognized non-motor feature of PD, but it has been less consistently characterized than olfactory loss, and its correlations with sex remain poorly explored. Recent clinical studies have reported a reduction in overall taste performance in PD across multiple modalities and cohorts [26,27]; some have even extended the assessment to include umami taste or intensity scaling, highlighting that chemosensory changes in PD are not limited to smell [33]. Our findings align with and extend these works by showing a marked reduction in total taste scores in PD and a specific male–female dissociation in the identification of the sour taste, with females outperforming males on citric acid. Female PD patients showed better recognition of the sour stimulus than males, while male PD patients were more likely to misidentify sour, an effect confirmed by the results from the CatBoost classifier, where poor recognition of citric acid pushed predictions toward male PD. These observations are consistent with the broader literature documenting non-motor sex differences in PD—females generally experiencing more prominent autonomic and affective symptoms, and males showing worse cognitive performance—underscoring the need for sex-aware phenotyping and stratification [30,31,34]. Taken together, these observations emphasize the need to include sex as a biological variable in the study of PD-related sensory dysfunction.

A particular novel aspect of our observations is the strong contribution of the tannic acid misidentification to female PD classification. Astringency is a complex oral-somatosensory perception arising when polyphenols (e.g., tannins) interact with salivary proteins—particularly proline-rich proteins (PRPs)—to reduce lubrication and increase friction, producing a drying/roughing mouthfeel [35,36]. This process involves both physicochemical precipitation and trigeminal/oral mechanosensory transduction and can vary with salivary flow, protein composition, age, and other host factors [37]. Our data suggest that PD-related sensory changes extend beyond canonical taste to include somatosensory dimensions such as astringency, and that these changes could be modulated by sex-specific biological factors, potentially related to the different protein composition of saliva and α-synuclein–related pathology.

In line with previous studies showing an increase in o-αsyn in the saliva of PD, while tot-αsyn is often reduced or unchanged [11,15,38,39], we showed an interaction of sex × belonging group for salivary levels of o-αsyn. Importantly, o-αsyn measurement discriminated the pathological from the healthy condition only in females. Moreover, unlike o-αsyn, tot-αsyn and p-αsyn showed no discriminatory value, suggesting that the observed effects are specific to the oligomeric species in this cohort. Finally, female PD patients who failed to recognize tannic acid showed the highest levels of o-αsyn. These findings add a sex-specific dimension and a functional correlate (misidentification of astringency), which could reflect differential peripheral α-syn pathology or salivary proteostasis in females. Although preliminary, our results suggest that peripheral α-synuclein biology might intersect with oral sensory function either through autonomic/secretomotor influences on salivary composition, by immune-inflammatory signaling affecting oral mucosa, or by more central gustatory network alterations that parallel α-syn pathology. These data are particularly relevant considering the recognized pathological role of oligomeric α-syn, suggesting that peripheral changes in this protein species may tightly reflect the central pathological process. In addition, the observation of higher levels of total salivary proteins in male PD patients versus male HCs, which was absent in females, further supports sex-dependent alterations at the oral milieu that may shape astringency perception via PRP–tannin interactions. Studies on the common *SNCA* SNPs have focused primarily on PD susceptibility and age of onset in populations [13,14,16,17,18], while less is known about how these variants modulate sensory phenotypes [28]. We found distinct associations between *SNCA* variants and the ability to perceive tannic acid in male and female PD patients for three of the four investigated SNPs—*rs356219*, *rs181489*, and *rs2583988.* These associations were not present for the other taste stimuli or in HCs, highlighting a possible selective relationship between certain SNCA SNPs and the impaired detection of astringency. Genotype distributions at the *rs356219* and *rs2583988* consistently distinguished individuals who correctly recognized tannic acid from those who did not in both males and females. In particular, the presence of the allele A (at *rs356219*) and the allele C (at *rs2583988*) characterized the M-yes and F-yes groups. In contrast, non-tasting PD patients (M-no, F-no) showed a predominance of allele G (at *rs356219*) and a more balanced distribution of CC and TT genotypes at *rs2583988*. These patterns suggest that specific SNCA variants may be linked to the preservation of taste function, while alternative alleles may contribute to sensory impairments. Notably, the *rs181489* SNP showed a sex-specific pattern. Significant differences were found only between F-yes and F-no, with female tasters showing a high frequency of allele C and female non-tasters showing enrichment for allele T. Sex-dependent genetic effects in PD have been previously suggested, particularly for *SNCA* variants, possibly reflecting hormonal modulation of αsyn expression or different patterns of neurodegeneration between males and females [40,41]. Since tannic acid causes an astringent sensation through interactions with salivary proteins and through trigeminal and gustatory pathways [42], our results support the emerging view that SNCA genetic variability may influence not only the classical motor aspects of PD, but also chemosensory processes. In addition, since αsyn plays a central role in synaptic homeostasis and neuronal plasticity [43], genetic variability modulating its expression or aggregation [44,45] could influence pathways responsible for the perception of more complex chemosensory stimuli such as astringency [28].

Some limitations should be considered. First, the sample sizes, although adequate for SL, could impact statistical power, especially for subgroup analyses, and may not fully capture the observed differences. Second, the study’s cross-sectional design precludes causal inferences or conclusions regarding relationships found between taste dysfunction, salivary levels of α-synuclein species, α-synuclein gene (*SNCA*) variants, sex, and PD. Although comparisons with HCs strengthen the validity of the study, longitudinal studies in larger cohorts will be needed to validate our findings. Third, even though the taste assessments were designed to minimize cognitive and educational biases, interferences from cognitive, socioeconomic, and educational variables, which have been shown to influence sensory performance [46,47], cannot be excluded. Fourth, salivary protein composition is known to be influenced by circadian rhythms [48]. Consequently, although saliva collection was standardized in our study, small variations cannot be eliminated. Finally, our results also showed that smoking status pushed the CatBoost model toward predicting PD in females. This should not be overinterpreted. Decades of epidemiological studies have shown a robust inverse association between smoking and PD risk [49,50,51,52]. The apparent discrepancy likely reflects cohort composition, granularity of smoking history, sex-specific sampling, or latent correlations (e.g., socioeconomic, dietary, or medication effects) rather than a true positive association.

## 4. Materials and Methods

### 4.1. Participants

A total of 159 participants were enrolled in the study and categorized into two groups: 99 PD patients (males, *n* = 60; females, *n* = 39) and 60 HCs (males, *n* = 32; females, *n* = 28) closely matched for age (range: 50–90 years) and ethnicity. PD patients were recruited at the Parkinson Center of the Department of Neurology, Brotzu Hospital, Cagliari, Italy, and HCs were recruited at the University of Cagliari, Italy. The exclusion criteria included food allergies, chronic rhinosinusitis, severe illnesses (such as diabetes and kidney disease), and use of drugs that impair sensory perception. PD patients were diagnosed according to Postuma criteria [53]. PD patients had no clinically significant cognitive deficits (indicated by a Mini-Mental State Examination (MMSE) score > 24) [54]. Their demographic and clinical features are shown in Table 1. Unfortunately, we were unable to determine the Hoehn and Yahr stage or disease type for 3 PD patients, and the age at initial diagnosis for 1. None of the HCs had first-degree relatives with neurodegenerative illnesses, and they were neurologically normal. A baseline medical screening was performed on the participants to inquire about their health status as well as anthropometric, demographic, and lifestyle characteristics, such as their smoking and dental hygiene practices. Two hours before the tests, participants were instructed not to consume any food, liquids, or tobacco products. They were examined for any lesions in the oral cavity, and no red blood cell contamination was found. Buccal swabs were collected and stored at −80° until molecular analyses were performed, and 3 mL of whole saliva samples was collected. Saliva samples were immediately treated with a protease inhibitor solution (cOmplete^®^, Roche Diagnostics, Monza, Italy). Saliva was collected from all participants at the same time in the afternoon to minimize potential variability related to circadian rhythm [48]. All participants were given a verbal explanation of the study and signed an informed consent form. The ethics committee of the University Hospital of Cagliari approved (Prot. PROFILE, verbal number 355, 13 March 2025) all procedures used in this investigation, which were carried out in compliance with the 1975 Declaration of Helsinki (updated in 2024).

### 4.2. Taste Perception Assessments

Taste perception of PD patients and HCs was determined through the correct identification of taste stimuli that were representative of the four basic taste qualities (sweet, sour, salty, bitter), and the sensation of astringency. These five taste assessments were applied to each participant according to Naciri et al. 2025 [28], by placing on his/her tongue taste strips (TST, Burghart Company, Wedel, Germany) impregnated with taste solutions, which were identified as those that most accurately can predict the overall taste status of HCs and patients with chemosensory loss [55]. Due to its persistence, the astringent taste stimulus was provided last in a semi-random order of presentation. Participants were asked to rinse their mouths with spring water before each test. The following solutions were used: 0.4 g/mL sucrose, 0.3 g/mL citric acid, 0.016 g/mL sodium chloride, 0.006 g/mL quinine hydrochloride, and 0.2 g/mL tannin. In a forced-choice procedure [56], participants had to identify, after each stimulation, the taste quality of each stimulus by choosing one of six possible answers (no taste, sweet, sour, salty, bitter, or astringent). Each correct answer was scored 1 point, and the number of correctly identified tastes was summed up in the total taste score, the maximum value of which was 5 for each participant. To minimize cognitive, socioeconomic, and educational biases, taste stimuli commonly experienced in everyday life were chosen and, to facilitate their identification, were presented together with written descriptors and representative images. The complete procedure required 10 min.

### 4.3. Salivary α-Synuclein Species Determination

The αsyn species (tot-αsyn, o-αsyn, and p-αsyn) were determined from saliva samples by performing enzyme-linked immunosorbent assays (ELISA).

After collection, saliva samples were treated with a protease inhibitor solution (cOmplete^®^, Roche Diagnostics). Briefly, one tablet was resuspended in 1.5 mL of DNA/RNA-free water according to the manufacturer’s instructions. The resulting solution was added to the saliva sample tube at a 1:7 (*v*/*v*) ratio (142.8 µL per 1 mL of saliva). The sample was immediately placed on ice to block the proteolytic activity. Samples were centrifuged for 20 min at 4 °C (5000× *g*) to remove any fragments or cell debris. The supernatant was separated from the pellet, and finally, the samples were stored at −80 °C until analysis.

Before performing ELISA, total protein content was quantified in all saliva samples using the Bicinchoninic Acid (BCA) Protein Assay Kit (Sigma-Aldrich, St. Louis, MO, USA). This step was carried out to normalize the levels of the different α-synuclein (α-syn) species and minimize potential variability attributable to differences in overall salivary protein concentration.

Frozen samples were thawed and centrifuged at 1000× *g* for 15 min at 4 °C before analysis. The concentrations of tot-αsyn, o-αsyn, and p-αsyn were then measured by ELISA following the manufacturer’s instructions. The following commercially available kits (MyBioSource, San Diego, CA, USA) were employed: Human Synuclein Alpha (SNCa) ELISA Kit (#MBS4502569) for total α-syn detection, Human Alpha Synuclein Oligomer (SNCOa) Sandwich ELISA Kit (#MBS730762) for oligomeric α-syn, and Human Phosphorylated Alpha Synuclein (PSNCA) ELISA Kit (#MBS038716) for phosphorylated α-syn. All assays had been previously validated for use in saliva through independent preliminary testing. According to the protocol by Angius et al. [11], samples were diluted 1:25 for the quantification of tot-αsyn and p-αsyn, and 1:2 for oligomeric o-αsyn.

Samples from both PD patients and HCs were analyzed in independent duplicate experiments. Optical density was measured at 450 nm using an Infinite M200 microplate reader (Tecan, Männedorf, Switzerland). Concentrations were calculated by interpolation from standard curves generated through regression analysis in Prism 8 (GraphPad Software, San Diego, CA, USA). Finally, the levels of each α-syn species were normalized to 1 mg of total protein for each individual saliva sample. Unfortunately, we were unable to measure the concentrations of the various αsyn species in some samples from PD patients (tot-αsyn, *n* = 4; o-αsyn, *n* = 13; p-αsyn, *n* = 5) and HCs (tot-αsyn, *n* = 2; o-αsyn, *n* = 4; p-αsyn, *n* = 2).

### 4.4. Genotyping of α-Synuclein Gene (SNCA) SNPs

Participants were genotyped for four single-nucleotide polymorphisms (SNPs) within the α-synuclein gene (*SNCA*): *rs356219* (A/G), whose allele G is linked to an increase in α-synuclein [13,14], *rs181489* (C/T), whose allele T is linked to a higher risk of developing PD [16], *rs2583988* (C/T), whose allele T is linked to a higher risk of developing PD and cognitive impairment [17,18], and *rs356186* (A/G), whose allele A is linked to a lower risk of developing PD [16].

DNA extraction and genotyping of SNCA SNPs were performed by using the salting-out method and the TaqMan^®^ SNP Genotyping Assay (Applied Biosystems, Life Technologies, Milan, Italy) according to Naciri et al. [28]. Briefly, DNA yield and purity were evaluated by measuring absorbance at 260 nm with a NanoDrop One/One Spectrophotometer (Thermo Fisher Scientific, Monza, Italy). Reactions were performed in 96-well plates under fast thermal cycling conditions. Each reaction included 10 ng of DNA, nuclease-free water, 1X TaqMan^®^ Genotyping Master Mix (code: 4371355), and 1X TaqMan^®^ Genotyping Assays (C_1020193_10, C_3208976_10, C_1658278_10, C_3208925_10). Amplification and fluorescence detection were carried out using the StepOne™ Real-Time PCR System, and genotypes were assigned by allelic discrimination analysis with Sequence Detection Software (version 2.3; Applied Biosystems). Each run included duplicate samples as well as positive and negative controls. We were unable to determine the genotype of some PD patients (2 for *rs356219*, 3 for *rs181489*, 5 for *rs2583988*, and 3 for *rs356186*) and HCs (2 for *rs356219*, 3 for *rs181489*, 3 for *rs2583988*, and 3 for *rs356186*) due to the poor concentration and/or purity of the extracted DNA.

### 4.5. Statistical Analysis

The distribution of male and female PD patients and male and female HCs who perceived no taste (under threshold), correctly recognized the taste (correct identification), or stated a different quality (incorrect identification) was compared for each taste stimulus (sucrose, NaCl, citric acid, quinine, and tannic acid) using Fisher’s exact test.

Differences between PD patients and HCs in the total taste scores were analyzed using the non-parametric Kruskal–Wallis test, followed by the Dwass–Steel–Critchlow–Fligner procedure, since the normality assessed by the Shapiro–Wilk test was violated. The same data were analyzed, divided by sex, using a Factorial General Linear Model with heteroscedasticity-consistent robust standard errors (HC3). This test ensures reliable inference under heteroscedasticity, deviations from normality, unequal variances, and unbalanced sample sizes, while preserving the interpretability of parameter estimates and marginal means. The Factorial General Linear Model was also used to compare the salivary levels of α-synuclein species (tot-αsyn, p-αsyn, and o-αsyn) in male and female PD patients and HCs and based on their ability to perceive taste stimuli. Post hoc comparisons were evaluated using Holm-adjusted *p*-values. Homogeneity assumptions were assessed using Levene’s test.

Fisher’s exact test (Genepop software version 4.8.5; https://cran.r-project.org/web/packages/genepop/index.html, accessed on 26 April 2026) was used to compare the genotype distributions and allele frequencies of the four SNPs (*rs356219*, *rs181489*, *rs2583988*, and *rs356186*) in male and female PD patients and HCs who correctly identified the taste (M-yes and F-yes) and those who did not perceive the taste or described a different quality (M-no and F-no).

Statistical analyses were performed using the Jamovi spreadsheet (version 2.6.44, The Jamovi Project, 2025, https://www.jamovi.org, accessed on 26 April 2026). The significance level was set at *p* ≤ 0.05.

### 4.6. Supervised Learning (SL)

By finding patterns that link input features to their corresponding labels, SL encompasses a class of algorithms that train models to predict labels for new samples using labeled datasets of previously recorded topic features. An automatic binary classification was carried out by using the CatBoost Classifier to clarify the diagnostic significance of taste stimuli, distinguishing between male and female PD patients and male and female HCs. This classifier learned the relationships between input features and target classes during training, allowing it to predict the correct class for new data according to Naciri et al. [28]. The features were as follows: Sucrose under threshold, Sucrose correct identification, Sucrose incorrect identification, NaCl under threshold, NaCl correct identification, NaCl incorrect identification, Citric acid under threshold, Citric acid correct identification, Citric acid incorrect identification, Quinine under threshold, Quinine correct identification, Quinine incorrect identification, Tannic acid under threshold, Tannic acid correct identification, Tannic acid incorrect identification, Total strips identified, Age, Smoker and Non-smoker. Categorical data were converted into a numerical format using the One-Hot Encoding technique [57], and the model performance was evaluated using common classification metrics, such as accuracy and macro-averaged F1-score [58]. The Synthetic Minority Oversampling Technique (SMOTE) was used to create synthetic samples for the minority class to solve the class imbalance in the training dataset, which had more PD patients than HCs [59]. To prevent bias and ensure generalization, the datasets were randomly split into training and testing sets in an 80:20 ratio for training models. We verified problems of overfitting by comparing the values of training accuracy with those of testing accuracy. Since they were strictly close to each other, the overfitting was excluded. In addition, we removed all features that were not significantly correlated with target classes and increased the regularization parameters. The Shapley Additive exPlanations (SHAP), a game-theoretical technique that links feature importance and feature effect, was used to interpret the SL model predictions [60]. It provides SHAP summary plots that link the influence and impact of features on the target class.

## 5. Conclusions

By integrating a multimodal design that includes sex stratification and the use of the SHAP algorithm for transparent data interpretation and concordance between classical statistics and ML, this work shows that sex is relevant to PD chemosensory phenotyping: salt, sour, and astringency have differential diagnostic weights in males and females; females with PD show a selective increase in salivary o-αSyn, and sex-sensitive SNCA–taste associations clearly emerge for astringency. These novel findings suggest that sex may modulate the interface between peripheral αsyn biology in oral tissues and oral sensory processing of both gustatory and astringent (tannin–saliva) cues. In females with PD, higher levels of the o-αsyn and altered salivary proteostasis may amplify disruptions in astringency transduction and recognition. In males, sour misidentification may be more prominent and linked to different peripheral or central pathways. *SNCA* common variants, associated with increased gene expression, may further modulate these phenotypes, producing the genotype-by-sex patterns we observed. The net result is a sex-specific sensory fingerprint that SL can harness to distinguish PDs from HCs. Replications with larger, more diverse cohorts and a deeper understanding of salivary biology are needed to translate these results into scalable clinical tools. In addition, future studies will focus on prospective clinical validation of these results and integration with more established markers before translation into neurological practice.

## Figures and Tables

**Table 1:** Demographic and clinical features of PD patients.

Features	Males	Females
sex (*n*)	60	39
Smokers/Non-smokers (*n*)	3/57	6/33
Age (*y*)	70.45 ± 1.23	69.07 ± 1.53
Age at onset (*y*)	62.50 ± 1.47	60.55 ± 1.85
Duration of illness (*y*)	7.95 ± 0.70	8.44 ± 0.89
Hoehn and Yahr stage	2.60 ± 0.16	2.73 ± 0.21
Type of PD:		
Tremor-dominant (*n*)	17	11
Akinetic-rigid (*n*)	7	7
Mixed (*n*)	36	19

Data are shown as the number of subjects or mean values ± S.E.

## Data Availability

Anonymized data underlying this article will be shared on reasonable request from any qualified investigator who wants to analyze questions that are related to the published article.

## Supplementary Materials

The following supporting information can be downloaded at: https://www.mdpi.com/article/10.3390/ijms27094048/s1.

## Abbreviations

The following abbreviations are used in this manuscript:

PD	Parkinson’s disease
HCs	healthy controls
M	male
F	female
*SNCA*	α-synuclein gene
αsyn	α synuclein
tot-αsyn	total α synuclein
o-αsyn	oligomeric α synuclein
p-αsyn	phosphorylated α synuclein
SL	supervised learning
SNPs	single-nucleotide polymorphisms
ELISA	enzyme-linked immunosorbent assay
BCA	bicinchoninic acid
HC3	robust standard errors
SHAP	Shapley Additive exPlanations
