# Supplementary material for: Sex-Specific Patterns of Taste Dysfunction, Their Relationships with α-Synuclein Profiling, and Supervised Learning-Based Diagnosis in Parkinson’s Disease (PD)
Source: Int J Mol Sci. 2026 Apr 30;27(9):4048. doi: 10.3390/ijms27094048 (PMC13164297; doi:10.3390/ijms27094048)
Supplement: Supplementary file 1 [file ijms-27-04048-s001.zip › ijms-4240658-supplementary.pdf]

Table S1. Genotype distribution and allele frequencies of polymorphisms of *SNCA* in the male and female PD patients and HCs.

	PD patients				*p*-values	HCs				*p*-values
	Males		Females			Males		Females		
	n	%	*n*	%		*n*	%	*n*	%	
rs356219										
Genotype										
GG	11	18.64	8	21.05	1.00	3	9.68	4	14.81	1.00
AG	30	50.85	18	47.37		18	58.06	12	44.44	
AA	18	30.51	12	31.58		10	32.26	11	40.74	
Allele										
G	52	44.07	34	44.74	1.00	24	38.71	20	37.04	1.00
A	66	55.93	42	55.26		38	61.29	34	62.96	
rs181489										
Genotype										
TT	8	14.04	6	15.38	0.765	2	6.67	4	14.81	0.697
CT	26	45.61	19	48.71		17	56.67	9	33.33	
CC	23	40.35	14	35.90		11	36.67	14	51.85	
Allele										
T	42	36.84	31	39.74	0.763	21	35.00	17	31.48	0.697
C	72	63.16	47	60.26		39	65.00	37	68.52	
rs2583988										
Genotype										
TT	8	13.79	5	13.89	0.880	2	6.45	3	11.54	0.838
CT	24	41.38	16	44.44		16	51.61	9	34..62	
CC	26	44.83	15	41.67		13	41.94	14	53.85	
Allele										
T	40	34.48	26	36.11	0.877	20	32.26	15	28.85	0.837
C	76	65.52	46	63.89		42	67.74	37	71.15	
rs356186										
Genotype										
AA	1	1.75	2	5.13	0.338	2	6.45	3	11.54	0.543
AG	15	26.32	12	30.77		12	38.71	11	42.31	
GG	41	71.93	25	64.10		17	54.84	12	46.15	
Allele										
A	17	14.91	16	20.51	0.334	16	25.81	17	32.69	
G	97	85.09	62	79.49		46	74.19	35	67.31	0.534

p-value derived from Fisher's method
